# Tiliroside Protects against Lipopolysaccharide-Induced Acute Kidney Injury via Intrarenal Renin–Angiotensin System in Mice

**DOI:** 10.3390/ijms242115556

**Published:** 2023-10-25

**Authors:** Xiaoli Yi, Chuanming Xu, Jing Yang, Chao Zhong, Huiru Yang, Le Tang, Shanshan Song, Jun Yu

**Affiliations:** 1 Translational Medicine Centre, Jiangxi University of Chinese Medicine, Nanchang 330002, China; yixiaoli@jxutcm.edu.cn (X.Y.); yangjing@jxutcm.edu.cn (J.Y.); 20181012@jxutcm.edu.cn (C.Z.); yanghuiru@jxutcm.edu.cn (H.Y.); tangle@jxutcm.edu.cn (L.T.); songshanshan@jxutcm.edu.cn (S.S.); 2 Center for Metabolic Disease Research, Department of Cardiovascular Sciences, Lewis Katz School of Medicine, Temple University, Philadelphia, PA 19140, USA; jun.yu@temple.edu

**Keywords:** Tiliroside, acute kidney injury, intrarenal RAS, ACE, ACE2

## Abstract

Tiliroside, a natural flavonoid, has various biological activities and improves several inflammatory diseases in rodents. However, the effect of Tiliroside on lipopolysaccharide (LPS)-induced acute kidney injury (AKI) and the underlying mechanisms are still unclear. This study aimed to evaluate the potential renoprotective effect of Tiliroside on LPS-induced AKI in mice. Male C57BL/6 mice were intraperitoneally injected with LPS (a single dose, 3 mg/kg) with or without Tiliroside (50 or 200 mg/kg/day for 8 days). Tiliroside administration protected against LPS-induced AKI, as reflected by ameliorated renal dysfunction and histological alterations. LPS-stimulated renal expression of inflammatory cytokines, fibrosis markers, and kidney injury markers in mice was significantly abolished by Tiliroside. This flavonoid also stimulated autophagy flux but inhibited oxidative stress and tubular cell apoptosis in kidneys from LPS-injected mice. Mechanistically, our study showed the regulation of Tiliroside on the intrarenal renin-angiotensin system in LPS-induced AKI mice. Tiliroside treatment suppressed intrarenal AGT, Renin, ACE, and Ang II, but upregulated intrarenal ACE2 and Ang1-7, without affecting plasma Ang II and Ang1-7 levels. Collectively, our data highlight the renoprotective action of Tiliroside on LPS-induced AKI by suppressing inflammation, oxidative stress, and tubular cell apoptosis and activating autophagy flux via the shift towards the intrarenal ACE2/Ang1-7 axis and away from the intrarenal ACE/Ang II axis.

## 1. Introduction

Acute kidney injury (AKI), characterized by a rapid decline of renal function, is a common clinical syndrome in about 50% of critically ill patients, which can subsequently progress to chronic kidney disease or end-stage renal disease [1]. AKI is one of the major risk factors for high in-hospital mortality and has become a global public health concern and an essential threat to human health [2], especially during the COVID-19 epidemic [3]. Sepsis-induced AKI, a common critical complication in intensive care units, caused 76% of in-hospital deaths [4]. It is well-known that enhanced inflammation, oxidative stress, immune cell infiltration, and renal tubular damage are involved in the pathological process of sepsis-induced AKI [4]. The current therapies for septic AKI, including vasopressor therapy, fluid resuscitation, and antibiotics, are nonspecific and reactive [4]. Thus, there is still a lack of specific and effective therapy strategies or drugs for septic AKI in clinical practice.

Since renin was first reported by Tigerstedt and Bergman in 1898 [5], the renin–angiotensin system (RAS) has been studied for nearly 125 years. Systemic RAS plays a pivotal role in multiple pathophysiological processes, including hypertension and kidney injury [6]. In addition to the well-known systemic RAS, the expression of multiple RAS components, including angiotensinogen (AGT), renin, angiotensin-converting enzyme (ACE), and angiotensin-converting enzyme 2 (ACE2), and the synthesis of angiotensin I (Ang I), Ang II, and Ang1-7, have been reported in the local tissues including the kidney [7]. Importantly, excessive activation of the intrarenal ACE/Ang II axis independently of the systemic RAS has been considered an essential mechanism of renal injury, whereas the activation of the intrarenal ACE2/Ang1-7 axis exhibits a renoprotective effect on renal injury [3,7]. Thus, targeting the balance between the ACE/Ang II and ACE2/Ang1-7 axis may be a good option for managing kidney injury.

Natural products, important sources of therapeutic agents, are collectively referred to as traditional Chinese medicine (TCM) in China and neighboring countries and have long been widely used to treat multiple human diseases, including AKI and its complications [8]. Among them, flavonoids, such as hispidulin [9], rutin [10], fisetin [11], and baicalin [12], are natural phenolic compounds commonly found in Chinese herbal medicines, vegetables, and fruits [13] and exert anti-inflammatory, antioxidant, and antiapoptotic actions, thus protecting against sepsis-induced AKI. Tiliroside (Tili) is a natural flavonoid commonly contained in multiple plants and has various biological activities, including anti-inflammatory and antioxidant activities [14]. Previous studies have shown that Tiliroside administration ameliorated depression [15], hypertension [16,17], cancer [18,19], osteoporosis [20], ulcerative colitis [21], and obesity–diabetes [22] in rodents. Although Tiliroside has been reported to exert an anti-inflammatory effect on lipopolysaccharide (LPS)-treated RAW 264.7 [23], mouse bone marrow-derived macrophages (BMDMs), and human THP-1 macrophages [21], the effect of Tiliroside on LPS-induced AKI and the underlying mechanisms have not been evaluated. This study aimed to determine the potential therapeutic effect of Tiliroside on LPS-induced AKI in mice.

## 2. Results

### 2.1. Tiliroside Ameliorated LPS-Induced Renal Dysfunction and Histological Abnormalities

**Figure 1:**
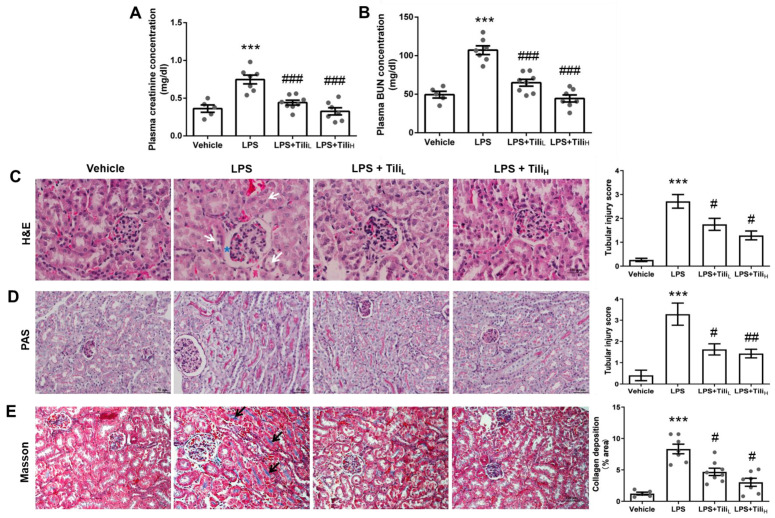
Effect of Tiliroside on kidney function and histological alterations in LPS-induced AKI mice. (**A**) Plasma creatinine concentration. (**B**) Plasma BUN concentration. (**C**) 24 h urinary albumin excretion. *n* = 5–8 in each group. (**C**) H&E staining. White arrows indicate detached tubular cells. Blue asterisks indicate dilated glomerular balloons. Scale bar: 100 μm. (**D**) Periodic Acid-Schiff (PAS) staining. Scale bar: 50 μm. (**E**) Masson staining. Black arrows indicate the positive area of collagen staining. Scale bar: 100 μm. *** *p* < 0.001 vs. Vehicle; ^#^ *p* < 0.05, ^##^ *p* < 0.01, and ^###^ *p* < 0.001 vs. LPS.

To assess renal function, we determined the two widely used indicators of kidney function, plasma creatinine and blood urea nitrogen (BUN) concentrations [24]. After LPS injection, the concentration of plasma creatinine (LPS, 0.75 ± 0.06 mg/dL vs. LPS + Tili_L_, 0.44 ± 0.03 mg/dL or LPS + Tili_H_, 0.33 ± 0.05 mg/dL, *p* < 0.001. Figure 1A) and BUN (LPS, 107.15 ± 5.67 mg/dL vs. LPS + Tili_L_, 64.90 ± 4.43 mg/dL or LPS + Tili_H_, 44.44 ± 4.56 mg/dL, *p* < 0.001. Figure 1B) were significantly elevated. Both low and high-dose Tiliroside administration attenuated these pathological effects. Hematoxylin and Eosin (H&E) (Figure 1C) and Periodic Acid–Schiff (PAS) staining (Figure 1D) were performed to assess the effect of Tiliroside on LPS-induced histological alterations in the kidney. LPS injection induced several histological alterations, including tubular cell detachment and glomerular balloon dilatation; these changes were attenuated by Tiliroside (Figure 1C,D). Masson staining for collagen revealed that LPS injection markedly increased collagen content in the kidney, and Tiliroside administration downregulated this change (Figure 1E).

### 2.2. Tiliroside Suppressed LPS-Induced Renal fibrosis

**Figure 2:**
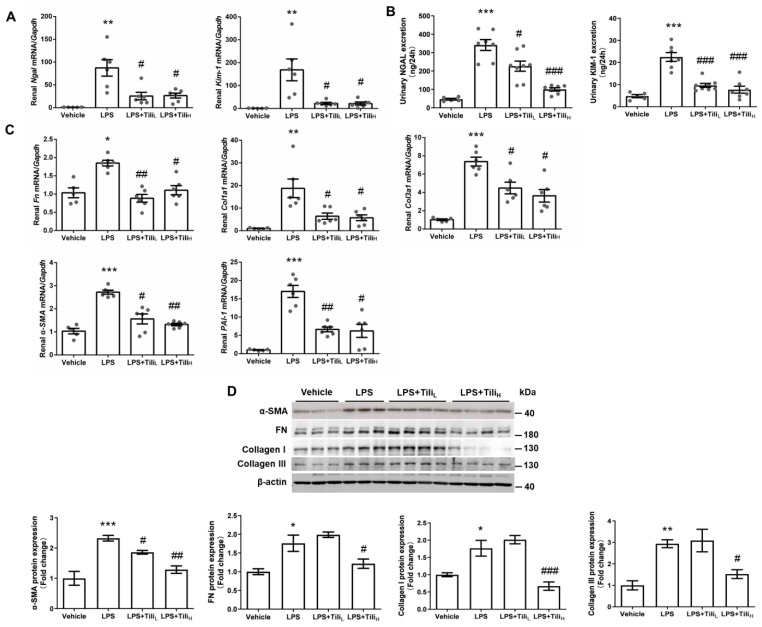
Effect of Tiliroside on renal fibrosis and injury in LPS-induced AKI mice. (**A**) Quantitative RT-PCR analysis of kidney injury genes, including *Ngal* and *Kim-1* mRNA expression in the kidneys with GAPDH as an internal control. (**B**) 24 h urinary NGAL and KIM-1 excretion assessed by ELISA. (**C**) Quantitative RT-PCR analysis of fibrosis genes, including *Col1a1*, *Col3a1*, *Fn*, *PAI-1*, and *α-SMA* mRNA expression in the kidneys with *Gapdh* as an internal control. (**D**) Representative immunoblotting and densitometric analysis of renal Collagen I, Collagen III, FN, and α-SMA protein expression with β-actin as an internal control. *n* = 5–6 in each group. * *p* < 0.05, ** *p* < 0.01, and *** *p* < 0.001 vs. Vehicle; ^#^ *p* < 0.05, ^##^ *p* < 0.01, and ^###^ *p* < 0.001 vs. LPS.

The renal mRNA expression of the typical tubular injury markers, including Neutrophil Gelatinase-associated Lipocalin (*Ngal*) and kidney injury molecule 1 (*Kim-1*) [25] assessed by RT-qPCR (Figure 2A), and the excretion of 24 h urinary NGAL and KIM-1 assessed by ELISA (Figure 2B), were significantly increased in LPS-injected mice, which were all attenuated by Tiliroside. Tiliroside also markedly inhibited the renal mRNA expression of various fibrosis markers, including fibronectin *(Fn)*, α-smooth muscle actin (*α-SMA*), collagen type I α1 (*Col1a1*), collagen type III α1 (*Col3a1*), and Plasminogen activator inhibitor-1 (*PAI-1*) in LPS-injected mice (Figure 2C). LPS-upregulated renal FN, α-SMA, Collagen I, and Collagen III protein expression was also consistently suppressed by high-dose Tiliroside treatment (Figure 2D).

### 2.3. Tiliroside Suppressed LPS-Induced Inflammation

**Figure 3:**
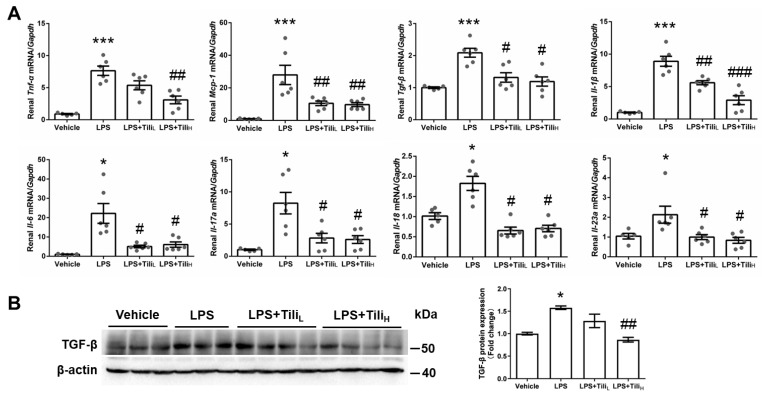
Effect of Tiliroside on renal inflammation in LPS-induced AKI mice. (**A**) Quantitative RT-PCR analysis of inflammatory genes, including *Tnf-α*, *Il-1β*, *Il-6*, *Il-17a*, *Il-18*, *Il-23a*, *Tgf-β*, and *Mcp-1* mRNA expression in the kidneys with *Gapdh* as an internal control. (**B**) Representative immunoblotting and densitometric analysis of renal TGF-β protein expression with β-actin as an internal control. *n* = 5–6 in each group. * *p* < 0.05 and *** *p* < 0.001 vs. Vehicle; ^#^ *p* < 0.05, ^##^ *p* < 0.01, and ^###^ *p* < 0.001 vs. LPS.

Inflammatory genes, such as *Tnf-α*, *Il-1β*, *Il-23a*, *Il-6*, *Il-17a*, *Il-18*, *Tgf-β*, and *Mcp-1*, were analyzed by using RT-qPCR. The mRNA levels of these genes in the kidney were markedly elevated in LPS-injected mice and blocked by Tiliroside treatment (Figure 3A). Using Western blotting, the renal expression of TGF-β protein was enhanced after LPS injection and significantly attenuated by the high dose of Tiliroside (Figure 3B).

### 2.4. Tiliroside Inhibited LPS-Induced Oxidative Stress

**Figure 4:**
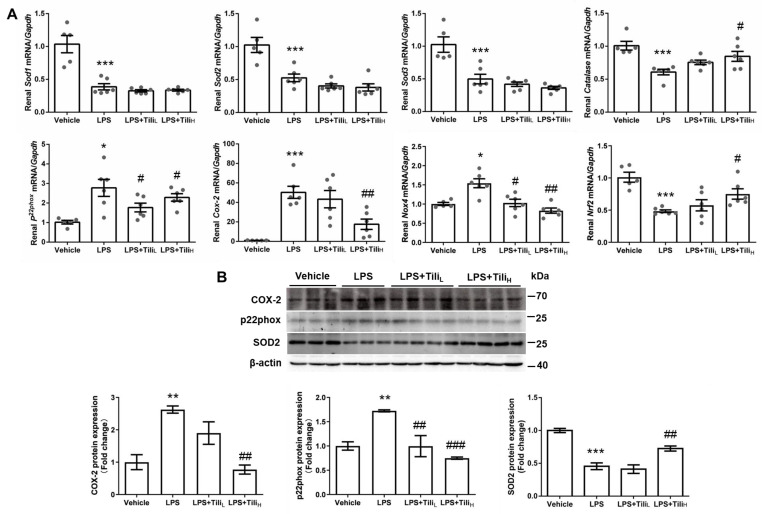
Effect of Tiliroside on renal oxidative stress in LPS-induced AKI mice. (**A**) Quantitative RT-PCR analysis of oxidative stress-related genes, including *Sod1*, *Sod2*, *Sod3*, *Catalase*, *Cox-2*, *p22phox*, *Nox4*, and *Nrf2* mRNA expression in the kidneys with *Gapdh* as an internal control. (**B**) Representative immunoblotting and densitometric analysis of renal COX-2, p22phox, and SOD2 protein expression with β-actin as an internal control. *n* = 5–6 in each group. * *p* < 0.05, ** *p* < 0.01, and *** *p* < 0.001 vs. Vehicle; ^#^ *p* < 0.05, ^##^ *p* < 0.01, and ^###^ *p* < 0.001 vs. LPS.

In LPS-injected mice, the *Catalase*, superoxide dismutase (*Sod*), and nuclear factor erythroid-2-related factor 2 (*Nrf2*) mRNA expression in the kidney was significantly downregulated, whereas *p22phox*, cyclooxygenase-2 (*Cox-2*), and NADPH oxidase 4 (*Nox4*) mRNA levels were markedly elevated (Figure 4A). Although Tiliroside administration did not affect the levels of *Sod1*, *Sod2*, and *Sod3* mRNA, high-dose of Tiliroside significantly reversed LPS-downregulated *Catalase* and *Nrf2* mRNA expression and inhibited LPS-stimulated *p22phox*, *Cox-2*, and *Nox4* mRNA expression (Figure 4A). Consistently, Tiliroside in high-dose attenuated LPS-induced renal p22phox and COX-2, and reversed LPS-downregulated renal SOD2 protein expression in LPS-injected mice (Figure 4B).

### 2.5. Tiliroside Enhanced Autophagic Flux but Not the Formation of Autophagosomes

**Figure 5:**
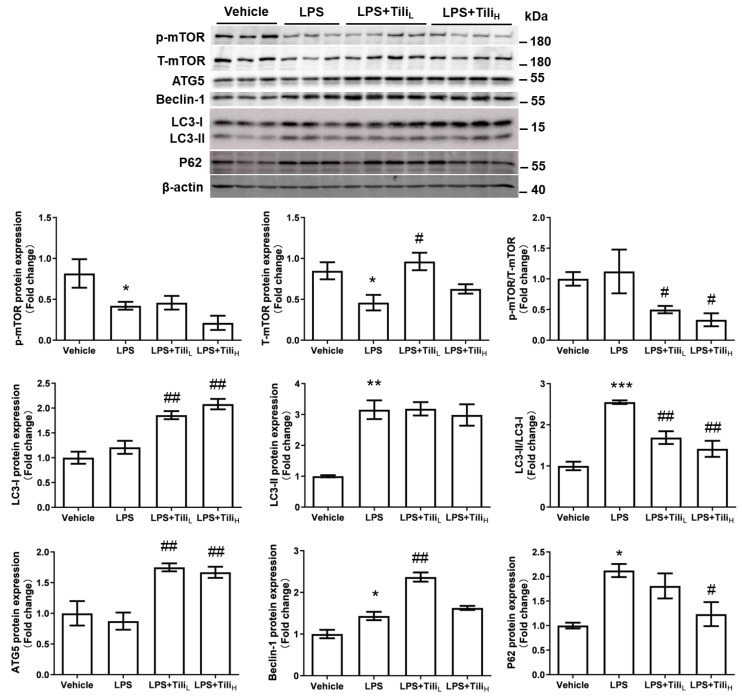
Effect of Tiliroside on autophagy in the kidneys in LPS-induced AKI mice. Representative immunoblotting and densitometric analysis of renal protein expression of autophagy markers, including mTOR, ATG5, Beclin-1, LC3, and P62 protein expression with β-actin as an internal control. *n* = 5–6 in each group. * *p* < 0.05, ** *p* < 0.01, and *** *p* < 0.001 vs. Vehicle; ^#^ *p* < 0.05 and ^##^ *p* < 0.01 vs. LPS.

The renal expression of autophagy-related proteins was detected by Western blotting. LPS injection slightly increased renal levels of Beclin-1, LC3-II, and P62 protein in mice (Figure 5), indicating the involvement of autophagy in LPS-induced AKI. ATG5, Beclin-1, and LC3-I protein levels but not LC3-II levels were further increased in mice with low-dose or high-dose Tiliroside treatment, accompanied by a reduced ratio of LC3-II/LC3-I. In contrast, only high-dose Tiliroside decreased P62 protein levels, indicating an enhanced autophagic flow by high-dose Tiliroside. We also detected the renal protein expression of the mammalian target of rapamycin (mTOR), a critical regulator of autophagy [26]. Although both total mTOR (T-mTOR) and phosphorylated (S2448) mTOR (p-mTOR) were decreased in the kidneys of LPS-injected mice, Tiliroside treatment reversed LPS-downregulated T-mTOR protein levels without affecting the levels of p-mTOR, resulting in the decreased p-mTOR/T-mTOR ratio, indicating the inhibition of mTOR by Tiliroside. These results suggest that Tiliroside enhanced autophagic flux but not the formation of autophagosomes in LPS-injected mice.

### 2.6. Tiliroside Abolished LPS-Induced Apoptosis

**Figure 6:**
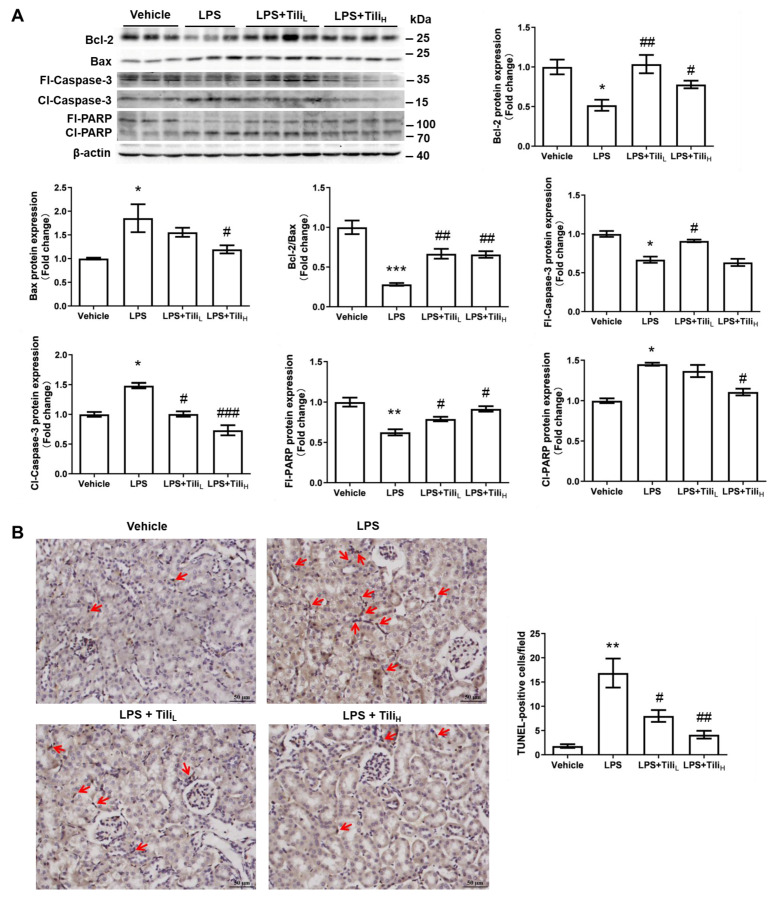
Effect of Tiliroside on apoptosis in the kidneys in LPS-induced AKI mice. (**A**) Representative immunoblotting and densitometric analysis of renal protein expression of apoptosis markers, including Bcl-2, Bax, Caspase 3, and PARP protein expression with β-actin as an internal control. (**B**) TUNEL staining and quantitative data of TUNEL-positive cells. Red arrows indicate the TUNEL-positive cells. Scale bar: 50 μm. *n* = 5–6 in each group. * *p* < 0.05, ** *p* < 0.01, and *** *p* < 0.001 vs. Vehicle; ^#^ *p* < 0.05, ^##^ *p* < 0.01, and ^###^ *p* < 0.001 vs. LPS.

We also detected the renal levels of apoptosis-related proteins using Western blotting. LPS injection decreased Bcl-2, full-length-caspase-3 (fl-caspase-3), and full-length poly ADP-ribose polymerase (fl-PARP) protein levels but increased Bax, cleaved caspase-3 (cl-caspase-3), and cleaved RAPA (cl-PARP) levels in the kidney (Figure 6A). All these changes were attenuated by Tiliroside treatment, especially the high-dose Tiliroside (Figure 6A), indicating the anti-apoptosis action of Tiliroside in LPS-injected mice. To further clarify this notion, apoptotic cells in kidney sections from LPS/Tiliroside-injected mice were examined using the TUNEL assay. LPS significantly increased the number of TUNEL-positive cells, which was markedly alleviated by Tiliroside (Figure 6B).

### 2.7. Tiliroside Regulated Intrarenal Renin-Angiotensin System

**Figure 7:**
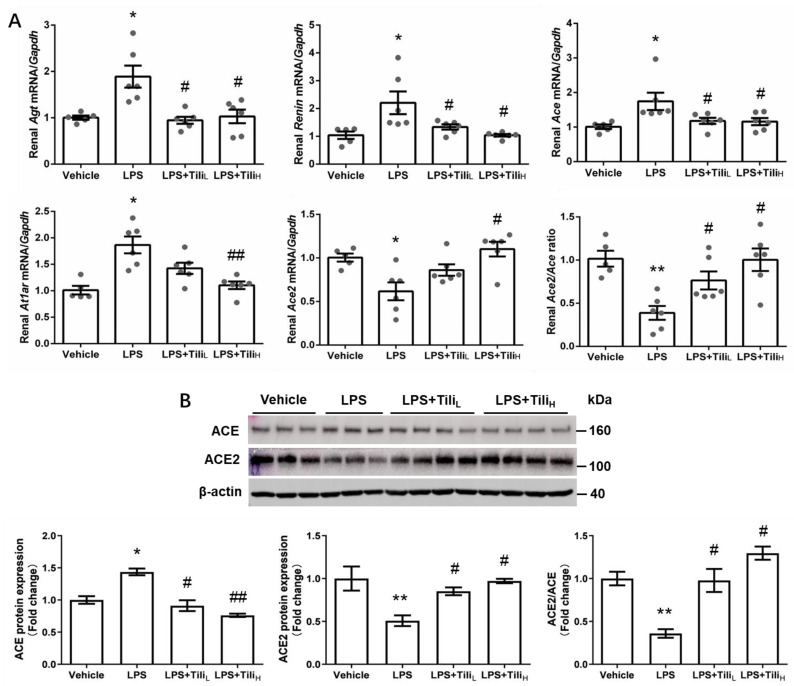
Effect of Tiliroside on the expression of intrarenal renin–angiotensin system components in LPS-induced AKI mice. (**A**) Quantitative RT-PCR analysis of *Agt*, *Renin*, *Ace*, *Ace2*, and *At1ar* mRNA expression in the kidneys with *Gapdh* as an internal control. (**B**) Representative immunoblotting and densitometric analysis of renal ACE and ACE protein expression with β-actin as an internal control. *n* = 5–6 in each group. * *p* < 0.05 and ** *p* < 0.01 vs. Vehicle; ^#^ *p* < 0.05 and ^##^ *p* < 0.01 vs. LPS.

It is well-known that the involvement of intrarenal RAS in the progress of AKI [3]. Thus, we assessed whether Tiliroside ameliorated LPS-induced AKI via modulating intrarenal RAS. Firstly, we detected the renal expression of RAS components using RT-qPCR and Western blotting. LPS injection significantly increased renal *Agt*, *Renin*, *Ace*, and *At1ar* mRNA levels and decreased renal *Ace2* mRNA levels, all of which were attenuated via Tiliroside administration (Figure 7A). Consistently, Tiliroside treatment abolished the LPS-induced upregulation of ACE protein expression and downregulation of ACE2 protein levels in mice, resulting in elevated ACE2/ACE ratio (Figure 7B), indicating that Tiliroside influences ACE/ACE2 balance.

**Figure 8:**
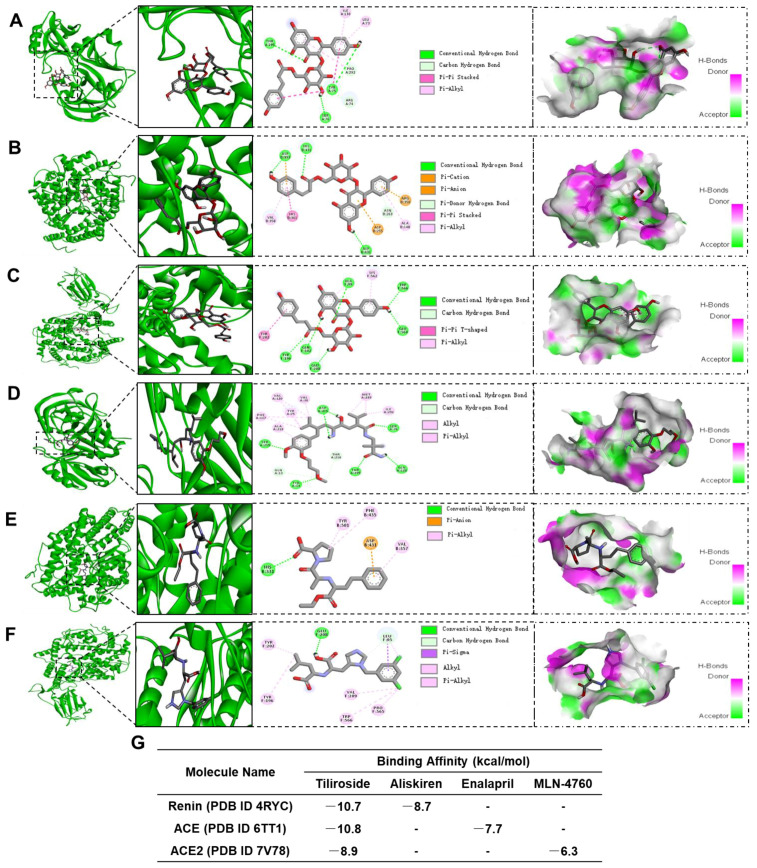
Molecular docking poses of Tiliroside with renin (PDB ID 4RYC) (**A**), ACE (PDB ID 6TT1) (**B**), and ACE2 (PDB ID 7V78) (**C**), aliskiren and renin (**D**), enalapril and ACE (**E**), MLN-4760 and ACE2 (**F**). (**G**) The binding affinity between Tiliroside and renin, ACE, and ACE, aliskiren and renin, enalapril and ACE, MLN-4760, and ACE2.

Secondly, we evaluated the potential interaction between Tiliroside and renin, ACE, and ACE2 protein using a molecular docking assay. As shown in Figure 8, similar to the high binding affinity between aliskiren and renin (Figure 8D), enalapril and ACE (Figure 8E), or MLN-4760 and ACE2 (Figure 8F), Tiliroside also shows high binding affinity to renin, ACE, and ACE2 (Figure 8A–C). The binding affinity between Tiliroside and renin, ACE, and ACE2 is −10.7 kcal/mol, −10.8 kcal/mol, and −8.9 kcal/mol, respectively. The predicted binding affinities are higher than that between aliskiren and renin (−8.7 kcal/mol), enalapril and ACE (−7.7 kcal/mol), or MLN-4760 and ACE2 (−6.3 kcal/mol) (Figure 8G).

**Figure 9:**
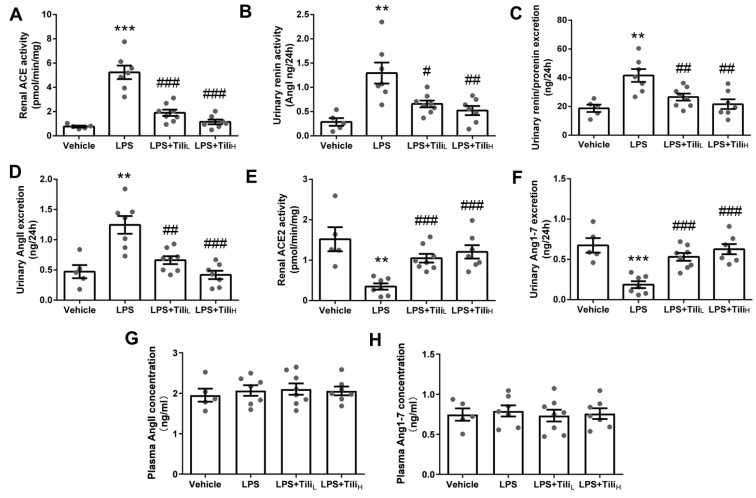
Effect of Tiliroside on renal ACE and ACE activity, urinary renin, Ang II, and Ang1-7 excretion, and plasma Ang II and Ang1-7 concentration in LPS-induced AKI mice. (**A**) Renal ACE activity. (**B**) Urinary renin activity. (**C**) 24 h urinary prorenin/renin excretion. (**D**) 24 h urinary Ang II excretion. (**E**) Renal ACE2 activity. (**F**) 24 h urinary Ang1-7 excretion. (**G**) Plasma Ang II concentration. (**H**) Plasma Ang1-7 concentration. *n* = 5–8 in each group. ** *p* < 0.01 and *** *p* < 0.001 vs. Vehicle; ^#^ *p* < 0.05, ^##^ *p* < 0.01, and ^###^ *p* < 0.001 vs. LPS.

Lastly, we measured the renal ACE and ACE2 activity, urinary renin activity, and urinary prorenin/renin, Ang II, and Ang1-7 excretion to further evaluate the regulation of Tiliroside on intrarenal RAS. LPS injection significantly elevated renal ACE activity (Figure 9A), urinary renin activity (Figure 9B), urinary prorenin/renin excretion (Figure 9C), and urinary Ang II excretion (Figure 9D), but decreased renal ACE2 activity (Figure 9E) and urinary Ang1-7 excretion (Figure 9F), which were significantly abolished by Tiliroside administration. However, there was no significant difference in plasma Ang II (Figure 9G) and Ang1-7 (Figure 9H) levels between the groups. Thus, these results indicated the modulating role of Tiliroside on the balance between the intrarenal ACE/Ang II axis and the intrarenal ACE2/Ang1-7 axis in LPS-induced AKI mice.

## 3. Discussion

**Figure 10:**
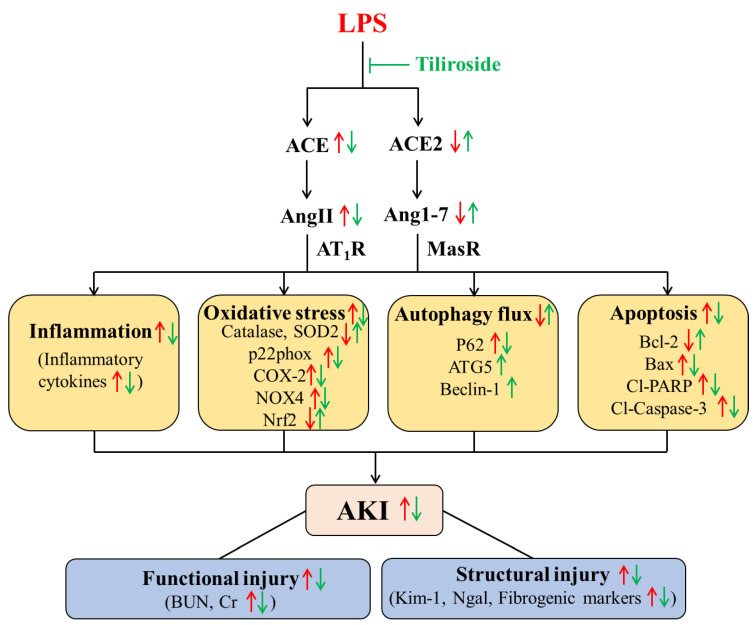
Schematic illustration of the key findings of the current study. Tiliroside ameliorated LPS-induced acute kidney injury by promoting autophagy and suppressing inflammation, apoptosis, and oxidative stress by modulating the intrarenal ACE/Ang II axis and ACE2/Ang1-7 axis balance.↑, Up-regulation or Promotion; ↓, Down-regulation or Inhibition.

Our present study aimed to investigate whether Tiliroside exerts a renoprotective action on endotoxin-induced AKI. Our data have demonstrated that the application of Tiliroside protected against LPS-induced AKI, as reflected by the reduction in plasma creatinine and BUN concentrations, as well as the inhibition of fibrosis, inflammation, apoptosis, and oxidative stress, and the stimulation of autophagy flux in the kidneys. Mechanistically, the renoprotective action of the Tiliroside may be associated with the shift towards the ACE2/Ang1-7 axis and away from the ACE/Ang II axis (Figure 10). Our findings may guide the use of Tiliroside in patients with AKI and call for clinical evaluation of the renoprotective properties of Tiliroside in patients with endotoxin-induced AKI.

Although several reports demonstrated the nephrotoxicity of some TCMs [27], accumulating evidence has shown the therapeutic potential of multiple TCM preparations or monomers for AKI mainly by suppressing inflammation and apoptosis and inhibiting oxidative stress [28]. Tiliroside, a natural glycosidic flavonoid, exerts multiple biological activities, including anti-inflammatory and anti-oxidant activities [14], thus exhibiting protective effect against various inflammatory diseases, such as neuroinflammation [29] and ulcerative colitis [21]. In the present study, Tiliroside administration significantly ameliorated LPS-induced functional and structural injury in the kidney of mice. On the one hand, we found that Tiliroside significantly decreased plasma creatinine and BUN levels and suppressed urinary albumin excretion, three widely used indicators for kidney function in animal experiments [24], indicating the beneficial effect of Tiliroside on LPS-induced renal dysfunction. On the other hand, Tiliroside alleviated renal structural injury, as reflected by a reduction in tubular cell detachment and glomerular balloon dilatation assessed by H&E staining, and the decreased mRNA expression of *Ngal* and *Kim-1*, two well-known tubular injury markers [25]. In addition, Tiliroside inhibited the renal accumulation of collagen and reduced the renal expression of various fibrosis markers, including FN, α-SMA, Collagen I, Collagen III, and PAI-1 in LPS-injected mice. Altogether, these results indicate a renoprotective action of Tiliroside on LPS-induced functional and structural injury.

Inflammation plays a vital role in the occurrence and development of AKI [4]. It is well-known that LPS injection can induce the overproduction of cytokines by stimulating several intracellular signaling pathways, including the NF-κB and MAPK pathways and the infiltration of immune cells (such as macrophages) into the injured tissues, thus contributing to the progression of endotoxin-induced AKI [4,9,30]. In the present study, LPS injection significantly stimulated the renal mRNA expression of inflammation-associated genes (*Tnf-α*, *Il-1β*, *Il-23a*, *Il-6*, *Il-17a*, *Il-18*, *Tgf-β*, and *Mcp-*1). Interestingly, Tiliroside treatment markedly attenuated the above inflammatory responses in mice. In agreement with our results, numerous previous studies have already reported the anti-inflammatory action of Tiliroside in different experimental inflammatory models [14,21,23,29,31,32]. Importantly, two studies have demonstrated that Tiliroside decreased cytokine production such as TNF-α and IL-6 in LPS-treated BV2 microglia cells (the resident macrophages of the brain [33]) [29,32], implying the inhibition effect of Tiliroside on inflammatory responses in LPS-treated macrophages. This was further supported by two studies from us [21] and others [23]. Jin et al. showed that Tiliroside inhibited LPS-inflammatory responses by suppressing the MAPK/JNK/p38 signaling in RAW 264.7 macrophage cells [23]. In an extension of this finding, our group recently reported that Tiliroside inhibited pro-inflammatory M1 macrophage polarization by blocking the HIF-1α/glycolysis pathway in the mouse BMDMs and human THP-1 macrophage cells [21]. In this regard, we reported that Tiliroside administration modulated the balance between pro-inflammatory M1 and anti-inflammatory M2 macrophages to ameliorate ulcerative colitis, as reflected by the decrease in the percentage of M1 macrophages (CD68^+^iNOS^+^) and the increase in the percentage of M2 macrophages (CD68^+^CD206^+^) in the colonic lamina propria of colitis mice [21]. Indeed, the infiltration of macrophages into the damaged tissue is increased in various inflammatory diseases, such as cisplatin or LPS-induced renal injury [9,34] and atherosclerosis [35]. Significantly, enhanced macrophage infiltration may exacerbate the LPS-induced inflammatory responses [4]. Therefore, we speculate that the suppression of macrophage infiltration in the kidney may contribute to the anti-inflammatory effect of Tiliroside in mice with LPS-induced AKI. However, the polarization status of macrophages in the kidney and whether Tiliroside ameliorates LPS-induced AKI through a macrophage-dependent manner is still unknown and future investigation is warranted.

Studies have shown that the stimulation of LPS on oxidative stress and tubular cell apoptosis in the kidneys plays essential roles in LPS-induced AKI [9,36,37]. In LPS-injected mice, the activity of antioxidant enzymes, including catalase and SODs, was suppressed in the kidney [9,37]. Similarly, we found that LPS significantly decreased renal *Catalase*, *Sod1*, *Sod2*, and *Sod3* mRNA expression. Although Tiliroside did not affect the mRNA expression of *Sod1*, *Sod2*, and *Sod3*, it reversed LPS-reduced *Catalase* mRNA expression at high doses. Of note, SOD2, an important scavenger of reactive oxygen species within the mitochondria, is mainly regulated post-translationally at protein levels [38]. Here, we found that decreased SOD2 protein levels in LPS mice were significantly reversed by Tiliroside. Thus, Tiliroside may inhibit oxidative stress by selectively regulating antioxidant enzymes. p22phox, an essential component of NADPH oxidase, and COX-2, an inducible enzyme expressed at sites of an inflammatory response, play a crucial role in oxidative stress [39,40]. The stimulation of NOX4-mediated reactive oxygen species production contributes to oxidative tissue damage in LPS-induced AKI [41]. We found that Tiliroside decreased LPS-stimulated renal p22phox, COX-2, and NOX4 expression in mice, especially in high-dose. Thus, suppressing the pro-oxidant and activating the antioxidant system may contribute to the antioxidant activity of Tiliroside in LPS-induced AKI. Furthermore, Nrf2, an important transcription regulatory factor of catalase and SODs, exhibits anti-inflammatory and anti-oxidant roles [42]. Previous studies have shown the renoprotective effect of activated Nrf2 on LPS-induced AKI [42,43]. Interestingly, Tiliroside has been shown to activate Nrf2 to suppress LPS/IFNγ-induced neuroinflammation and oxidative stress in BV2 microglia cells [29]. Similarly, LPS-reduced renal Nrf2 expression was significantly abolished by high-dose Tiliroside in mice. Thus, Tiliroside stimulated catalase, possibly by activating Nrf2 in the kidneys of LPS-injected mice. Additionally, we, for the first time, suggest the antiapoptotic effect of Tiliroside under inflammatory conditions. We found that Tiliroside significantly suppressed tubular cell apoptosis in LPS-injected mice, as reflected by the increased Bcl-2 expression, decreased Bax expression, the reversal of PARP and Caspase-3 activation, and the reduced TUNEL-positive cells. Therefore, Tiliroside ameliorates LPS-induced AKI, at least in part, through its anti-oxidant and anti-apoptotic actions.

Autophagy, an adaptive catabolic process, plays an essential role in various physiopathological processes, including water deprivation-induced antidiuretic [44] and sepsis-induced AKI [45,46]. Of note, the autophagy levels in the kidney under sepsis remain controversial. In sepsis-induced AKI, 58.5% of the studies reported the activation of autophagy, while 19.5% of the reports showed the inhibition of autophagy, and 22.0% of the studies showed that autophagy was activated in the early stage but suppressed in the later phase [46]. However, accumulated evidence has demonstrated autophagy’s critical cytoprotective role in sepsis-induced AKI [46]. In the present study, LPS injection slightly increased renal Beclin-1 and p62 protein expression and the ratio of LC3-II/LC3-I, suggesting the possible enhancement of autophagosome formation and inhibition of autophagy flux in these mice. For the first time, we reported the regulation of autophagy by Tiliroside in the kidney of LPS-injected mice. Tiliroside administration increased ATG5 and Beclin-1 protein expression and abolished LPS-upregulated p62 protein expression in mice kidneys, without affecting the levels of LC3-II. These results suggest that Tiliroside improved LPS-induced AKI in mice possibly by enhancing autophagy flux but not the formation of autophagosomes. Further studies are needed to clarify whether the renoprotection action of Tiliroside is dependent on the enhanced autophagy flux. mTOR, a key inhibitory regulator of autophagy, plays a critical biological function in sepsis-induced AKI [47]. Although LPS reduced the renal levels of T-mTOR and p-mTOR (S2448), the ratio of p-mTOR/T-mTOR was maintained in mice. Interestingly, Tiliroside treatment decreased the ratio of p-mTOR/T-mTOR by upregulating T-mTOR levels without affecting the levels of p-mTOR. These results may suggest that Tiliroside stimulated autophagy by inhibiting the mTOR pathway. However, the possible mechanism of Tiliroside regulating mTOR is unclear and awaits future investigation.

Accumulating studies have demonstrated the existence of intrarenal RAS, which was recognized as an essential mechanism for the pathogenesis of renal disease and hypertension [3,7,48,49]. Importantly, it is well-known that the imbalance between the intrarenal ACE/Ang II and ACE2/Ang1-7 axis contributes to kidney injury [3]. Specifically, the intrarenal ACE/Ang II axis exhibits injury-promotion effects on AKI. In contrast, the intrarenal ACE2/Ang1-7 axis counters the effect of the ACE/Ang II axis and exerts renoprotective actions on AKI [3]. Herein, we hypothesized that the imbalance between the intrarenal ACE/Ang II and ACE2/Ang1-7 axis served as a molecular mechanism for the renoprotective action of Tiliroside in LPS-injected mice. Along this line, we found that Tiliroside significantly suppressed the intrarenal ACE/Ang II axis but activated the intrarenal ACE2/Ang1-7 axis in LPS-induced AKI. First, Tiliroside inhibited the LPS-stimulated expression of *Agt*, *Renin*, *Ace*, and *At1ar* mRNA and ACE protein in the kidney and attenuated LPS-reduced renal *Ace2* mRNA and ACE2 protein expression. Second, we found that Tiliroside exerts high binding affinity to renin, ACE, and ACE2, assessed by molecular docking assay. Third, Tiliroside treatment markedly blocked LPS-stimulated renal ACE activity, urinary renin activity, and urinary prorenin/renin excretion but abolished LPS-reduced renal ACE2 activity, resulting in decreased urinary Ang II excretion and increased urinary Ang1-7 excretion, accompanied with the improved AKI. Consistent with the previous reports [50,51], we found that the levels of plasma Ang II and Ang1-7 were unchanged in LPS-injected mice with or without Tiliroside treatment. Overall, Tiliroside functions as a renoprotective factor in LPS-induced AKI, at least in part, depending on the inhibition of the intrarenal ACE/Ang II axis and the activation of the intrarenal ACE2/Ang1-7 axis, independently of the systemic RAS.

The present study has a number of limitations. A major one is that the precise target of Tiliroside’s renoprotective action is still unknown. Although this problem is commonly encountered in TCM research employing TCM preparations or monomers, modern biotechnologies, including RNA sequencing and proteomics, should be applied to future TCM studies to identify the key target of Tiliroside. In addition, the oral bioavailability of flavonoids is usually low due to their poor aqueous solubility, which limits or hinders their clinical application [52]. Indeed, the Tiliroside also exhibits low bioavailability regulated by multidrug resistance-associated protein 2 (MRP2) -influenced intestinal absorption of the Tiliroside [53]. In the present study, Tiliroside was water-insoluble and administrated by gavage, which may limit its full biological effects. Thus, it is essential to develop strategies to enhance the oral bioavailability of Tiliroside, for example, by enhancing its solubility [54] and inhibiting MRP2 to improve its intestinal absorption [53].

## 4. Materials and Methods

### 4.1. Animal Care

All C57BL/6 mice (Hunan SJA laboratory animal Co., Ltd., Changsha, China) were given free access to tap water and were fed the standard diet. Mice were housed in a temperature- and humidity-controlled room with a 12:12 h light-dark cycle. All animal studies were conducted with the approval of the Jiangxi University of Chinese Medicine Animal Care and Use Committee (No. 20230043) in accordance with the National Institutes of Health Guide for the Care and Use of Laboratory Animals.

### 4.2. Animal Treatment

**Figure 11:**
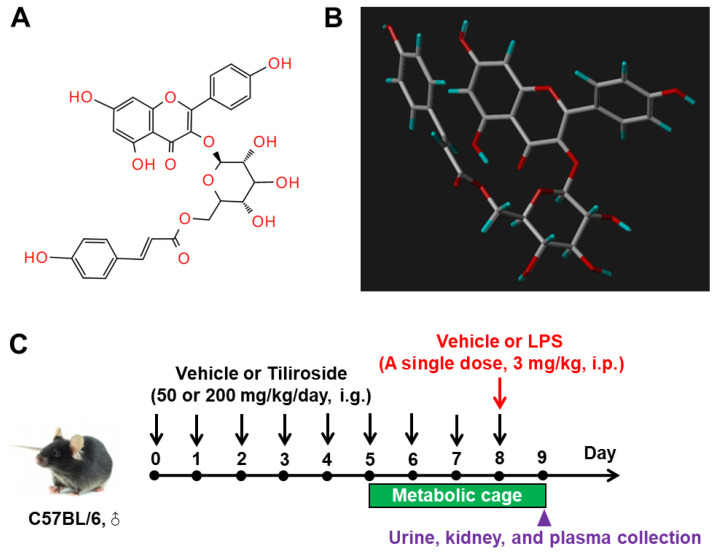
The 2D (**A**) and 3D (**B**) chemical structure of Tiliroside and experimental procedure for lipopolysaccharide (LPS)-induced acute kidney injury in C57BL/6 mice and Tiliroside administration (**C**).

Male C57BL/6 mice (25–30 g) were divided into four groups (Vehicle, LPS, LPS + Tili_L_, and LPS + Tili_H_). Mice were housed in metabolic cages for 4 days in a temperature- and humidity-controlled room with a 12:12 h light–dark cycle for 24 h urine collection. The LPS, LPS + Tili_L_, and LPS + Tili_H_ mice received a single intraperitoneal injection of LPS (3 mg/kg, dissolved in saline). The LPS + Tili_L_ and LPS + Tili_H_ mice were pretreated with Tiliroside (purity > 98%, Chengdu Desite Bio Technology, Chengdu, China) (Tili_L_: 50 mg/kg/day; Tili_H_: 200 mg/kg/day, dissolved in 10% DMSO and 90% corn oil) (Figure 11A,B) via gavage for 8 days and followed by LPS treatment for 24 h. The same volumes of vehicles were given to the mice in the Vehicle or LPS group. The experimental procedure is summarized in Figure 11C. 24 h urine samples were collected from 8:30 to 9:00 a.m. on day 9. At 24 h after LPS injection, all mice were anesthetized and sacrificed, and blood and kidney samples were harvested for further analyses.

### 4.3. Biochemical Analyses of Plasma and Kidney Tissues

The plasma creatinine and blood urea nitrogen (BUN) concentrations were measured using a Creatinine Assay kit (C011-2-1) and Urea Assay Kit (C013-1-1) (Nanjing Jiancheng Biological Engineering Research Institute, Nanjing, China), respectively. Renin activity in urine was determined by the delta value of the AngI generation using an AngI EIA kit (S-1188, BMA Biomedicals, Augst, Switzerland) from the sample incubating at 4 °C and 37 °C for 1 h, respectively [55,56]. Total renin/prorenin, Ang II, Ang1-7, albumin, NGAL, and KIM-1 released into the urine were measured by ELISA assay using a total mouse renin/prorenin ELISA kit (MPRENKT-TOT, Molecular Innovations, Novi, MI, USA), Ang II ELISA kit (ADI-900-204, Enzo Life Sciences, Farmingdale, NY, USA), mouse Ang1-7 ELISA kit (EM1634, Wuhan Fine Biotech Co., Ltd., Wuhan, China), mouse NGAL ELISA KIT (SEKM-0119, Solarbio, Beijing, China), and mouse KIM-1 ELISA KIT (SEKM-0147, Solarbio, Beijing, China), respectively. The ACE activity in homogenates of renal tissues was determined by a fluorometric method as described previously [57], and the ACE2 activity was measured by using the ACE2 Activity Fluorometric Assay Kit (P0319S, Beyotime Biotechnology, Shanghai, China), values were normalized by the total protein content of the tissue. All analyses were carried out following the manufacturers’ instructions.

### 4.4. Quantitative Reverse Transcriptase PCR (RT-qPCR)

Snap-frozen renal samples were homogenized in TRIzol reagent (15596018, Life Technologies, Carlsbad, CA, USA). Extracted RNA was reverse transcribed into cDNA using the HiScript Q RT SuperMix (R122, Vazyme, Nanjing, China). Quantitative PCR was performed using the Hieff^®^ qPCR SYBR Green Master Mix reagent (11201ES, Yeasen, Shanghai, China) and specific primer (Table 1) in the LightCycler^®^ 96 System (Roche, Ricardo Rojas, Argentina). All reactions were run in duplicate. All experiments were carried out following the manufacturers’ protocols. Relative mRNA expression levels were calculated from threshold cycle numbers (CT), i.e., 2^−ΔΔCT^, according to the manufacturer’s suggestion. The data were shown as a relative value normalized by *Gapdh*.

### 4.5. Western Blotting (WB)

Renal tissues were lysed and subsequently sonicated in homogenization buffer (0.3 M sucrose, 50 mM Tris-HCl, 1 mM EDTA, 1 mM EGTA, 1 mM DTT, pH 7.5) with 1 mM protease inhibitor cocktail (Roche, Berlin, Germany) [55,56]. According to the manufacturer’s instructions, protein concentrations were determined using the Pierce BCA Protein Assay Kit (Thermo Scientific, Rockford, IL, USA). Samples were resolved by SDS-PAGE and transferred onto a polyvinylidene fluoride membrane (Immobilion-P, Millipore, Bedford, MA, USA). The membranes were blocked with 5% bovine serum albumin [BSA] in Tris-buffered saline with Tween-20 (TBST) for 1 h at room temperature, followed by overnight incubation with primary antibodies (Table 2) diluted in antibody dilution buffer (1.5 g BSA, 0.1 g NaN_3_, 50 mL TBST) at 4 °C. After washing with TBST, membranes were incubated with secondary antibodies (IRDye^®^ 800CW Goat anti-Mouse Antibody, IRDye^®^ 680LT Goat anti-Rabbit Antibody) (LI-COR, Lincoln, NE, USA) for 1 h at room temperature. Signals on immunoblots were detected using Odyssey System (OSA-0323, LI-COR, Lincoln, NE, USA) and quantitated using Image-Pro Plus version 6.0 software. Values were normalized to the mean intensity measured in the control groups. The relative expression of the protein of interest was normalized to loading control β-actin.

### 4.6. Histological Examination and Immunofluorescence Staining

Kidneys were fixed overnight at 4 °C in 4% paraformaldehyde and processed for histological examination and immunofluorescence analysis. Paraffin-embedded tissue samples were cut into 3-μm-thick sections, deparaffinized, and rehydrated. Slides were used for H&E, Masson, or PAS staining for histological examination according to the manufacturers’ instructions. The kidney tubular damage score corresponded to the following percentages of kidney tubular damage: 0, 0%; 1, ≤10%, 2, 11–25%; 3, 26–45%; 4, 46–75%; and 5, 76–100% based on tubular cell necrosis, tubular dilation, cast formation, and the loss of brush border [58]. Apoptotic cells in kidney sections were determined using a Biotin TUNEL assay kit (T2191, Solarbio, Beijing, China) according to the manufacturer’s instructions. Images were captured using a Leica DMI4000B fluorescence microscope (Wetzlar, Germany). The quantification of positive staining was analyzed using Image-Pro Plus version 6.0 software.

### 4.7. Molecular Docking Assay

The chemical structures of Tiliroside, aliskiren, enalapril, and MLN-4760 were drawn using ChemDraw20.0, optimized using the Avogadro Application 1.2.0 to correct the geometry configuration [59], and saved in PDB file format for molecular docking analysis. The crystal structures of renin (PDB ID 4RYC), ACE (PDB ID 6TT1), and ACE2 (PDB ID 7V78) were obtained from RSCB protein database, evaluated using PyMOL molecular graphics system software (https://pymol.org/, PyMOL v2.4.2, accessed on 1 January 2023) to remove the surrounding water, ligands, and other molecules, and then hydrogenated using AutoDock Tools, and saved in PDBQT file format [60]. The docking analysis was performed using AutoDock VINA software (v1.1.2) [61].

### 4.8. Statistical Analysis

Data are summarized as means ± SEM. Statistical analysis was performed using one-way variance analysis (ANOVA) with the Bonferroni test for multiple comparisons using IBM SPSS 19 software (SPSS 19 v1.0.0). The difference was considered significant when the probability value was less than 0.05.

## Figures and Tables

**Table 1:** Primers used for RT-qPCR of gene expression.

Gene	Sequence (5′→3′)
*Ngal*	(F) GCAGGTGGTACGTTGTGGG
	(R) CTCTTGTAGCTCATAGATGGTGC
*Fn*	(F) ATGTGGACCCCTCCTGATAGT
	(R) GCCCAGTGATTTCAGCAAAGG
*Col1a1*	(F) TAAGGGTCCCCAATGGTGAGA
	(R) GGGTCCCTCGACTCCTACAT
*Col3a1*	(F) CTGTAACATGGAAACTGGGGAAA
	(R) CCATAGCTGAACTGAAAACCACC
*α-SMA*	(F) CCCAGACATCAGGGAGTAATGG
	(R) TCTATCGGATACTTCAGCGTCA
*Kim-1*	(F) AGCAGTCGGTACAACTTAAAGG
	(R) ACTCGACAACAATACAGACCAC
*PAI-1*	(F) TCTGGGAAAGGGTTCACTTTACC
	(R) GACACGCCATAGGGAGAGAAG
*Tnf-α*	(F) CCCTCACACTCAGATCATCTTCT
	(R) GCTACGACGTGGGCTACAG
*Mcp-1*	(F) TTAAAAACCTGGATCGGAACCAA
	(R) GCATTAGCTTCAGATTTACGGGT
*Tgf-β*	(F) TACGCCTGAGTGGCTGTCTT
	(R) CGTGGAGTTTGTTATCTTTGCT
*Il-6*	(F) TAGTCCTTCCTACCCCAATTTCC
	(R) TTGGTCCTTAGCCACTCCTTC
*Il-17a*	(F) TCAGCGTGTCCAAACACTGAG
	(R) CGCCAAGGGAGTTAAAGACTT
*Il-18*	(F) GTGAACCCCAGACCAGACTG
	(R) CCTGGAACACGTTTCTGAAAGA
*Il-1β*	(F) GAAATGCCACCTTTTGACAGTG
	(R) TGGATGCTCTCATCAGGACAG
*Il-23a*	(F) ATGCTGGATTGCAGAGCAGTA
	(R) ACGGGGCACATTATTTTTAGTCT
*Sod1*	(F) AACCAGTTGTGTTGTCAGGAC
	(R) CCACCATGTTTCTTAGAGTGAGG
*Sod2*	(F) TGGACAAACCTGAGCCCTAAG
	(R) CCCAAAGTCACGCTTGATAGC
*Sod3*	(F) CCTTCTTGTTCTACGGCTTGC
	(R) GCGTGTCGCCTATCTTCTCAA
*Catalase*	(F) AGCGACCAGATGAAGCAGTG
	(R) TCCGCTCTCTGTCAAAGTGTG
*p22phox*	(F) AGCGATGTGGACAGAAGTACC
	(R) CAGCCCGGACGTAGTAATTCC
*Cox-2*	(F) TGCACTATGGTTACAAAAGCTGG
	(R) TCAGGAAGCTCCTTATTTCCCTT
*Nrf2*	(F) GCCCACATTCCCAAACAA
	(R) TGTCCTGCTCTATGCTGCT
*Nox4*	(F) TGTTGGGCCTAGGATTGTGTT
	(R) AGGGACCTTCTGTGATCCTCG
*Agt*	(F) TGTGACAGGGTGGAAGATGA
	(R) AGATCATGGGCACAGACACC
*Renin*	(F) GTGACTGTGGGTGGAATCACTGT
	(R) GCCAGCATGAAAGGGATCAG
*Ace*	(F) TTGCTATGGGCATGGAAGAG
	(R) CAGGTCTTGCTCCAGGTTGT
*Ace2*	(F) ACTACAGGCCCTTCAGCAAA
	(R) TGTCGCCATTATTTCATCCA
*At1ar*	(F) AACTCACAGCAACCCTCCAA
	(R) ATCACCACCAAGCTGTTTCC
*Gapdh*	(F) AGGTCGGTGTGAACGGATTTG
	(R) TGTAGACCATGTAGTTGAGGTCA

**Table 2:** Antibodies used for Western blotting.

Antibodies	Identifier	Source	Dilution
Anti-β-Actin	TA811000	ORIGENE	1:2000
Anti-α-Smooth Muscle Actin	19245T	CST	1:1000
Anti-FN/Fibronectin	WL03677	Wanaleibio	1:500
Anti-Collagen I	WL0088	Wanaleibio	1:500
Anti-Collagen III	WL03186	Wanaleibio	1:500
Anti-TGF β1	ab179695	Abcam	1:1000
Anti-COX2	ab179800	Abcam	1:1000
Anti-p22-phox	WL03514	Wanaleibio	1:500
Anti-mTOR	T55306F	Abmart	1:1000
Anti-Phospho-mTOR(S2448)	T56571F	Abmart	1:500
Anti-ATG5	T55766F	Abmart	1:1000
Anti-Beclin 1	11306-1-AP	Proteintech	1:1000
Anti-LC3b	2775s	CST	1:1000
Anti-p62	18420-1-AP	Proteintech	1:1000
Anti-Bcl-2	15071S	CST	1:1000
Anti-Bax	14796S	CST	1:1000
Anti-Caspase-3/Cleaved Caspase-3	WL02117	Wanaleibio	1:500
Anti-PARP/Cleaved-PARP	WL01932	Wanaleibio	1:300
Anti-ACE	ab39172	Abcam	1:1000
Anti-ACE2	ab15348	Abcam	1:1000
Anti-SOD2	WL02506	Wanaleibio	1:500

## Data Availability

The datasets created during and/or analyzed during the current study will be available from the corresponding author upon reasonable request. There is no security, licensing, or ethical issues related to these data.
